# Supplementary material for: Learning nonequilibrium statistical mechanics and dynamical phase transitions
Source: Nat Commun. 2024 Feb 6;15:1117. doi: 10.1038/s41467-024-45172-8 (PMC10847122; doi:10.1038/s41467-024-45172-8)
Supplement: Supplementary file 1 — SUPPLEMENTARY INFO [file 41467_2024_45172_MOESM1_ESM.pdf]

Supplementary Information: Learning nonequilibrium statistical mechanics and dynamical phase transitions

Ying Tang,[1,2,*] Jing Liu,[3,4,†] Jiang Zhang,[4,5] and Pan Zhang[3,6,7,‡]

[1] *Institute of Fundamental and Frontier Sciences, University of Electronic Sciences and Technology of China, Chengdu 611731, China*
[2] *International Academic Center of Complex Systems, Beijing Normal University, Zhuhai 519087, China*
[3] *CAS Key Laboratory for Theoretical Physics, Institute of Theoretical Physics, Chinese Academy of Sciences, Beijing 100190, China*
[4] *School of Systems Science, Beijing Normal University, Beijing 100875, China*
[5] *Swarma Research, Beijing 102308, China*
[6] *School of Fundamental Physics and Mathematical Sciences, Hangzhou Institute for Advanced Study, UCAS, Hangzhou 310024, China*
[7] *Hefei National Laboratory, Hefei 230088, China*

CONTENTS

I. A reinforcement-learning approach to track nonequilibrium dynamics 2

II. Evaluating the dynamical partition function 3
A. The method of projection 4

III. Enhancement on tracking the phase transition 4
A. Importance sampling 4
B. Proper choices on the initialization of the VAN 5

IV. Voter model in two dimension 5

V. Kinetically constrained models 6
A. One-dimensional cases 6
B. Two-dimensional cases 6
C. Three-dimensional cases 7
D. Computational details for kinetically constrained models 7

VI. Supplementary Figures 9

References 16

* These authors contributed equally; Corresponding authors: jamestang23@gmail.com
† These authors contributed equally
‡ Corresponding authors: panzhang@itp.ac.cn

In this Supplementary Information, we demonstrate the algorithm of tracking time evolution of the probability distribution by the variational autoregressive network (VAN) and learning the dynamical partition function. We also provide details of tracking the phase transition. We further give the information on applying the approach to the voter model and kinetically constrained models (KCMs). The mathematical derivation of the Supplementary Information is in a self-consistent manner.

I. A REINFORCEMENT-LEARNING APPROACH TO TRACK NONEQUILIBRIUM DYNAMICS

To track time evolution under the tilted generator $\mathbb{W}_s$ for a small time step $\delta t = t_{j+1} - t_j \ll 1$, we use the Suzuki-Trotter decomposition [1]. Given the probability distribution $\hat{P}_j^{\theta_j}(\mathbf{x})$ (the hat symbol denotes that the probability is parameterized by the neural network model) at the current time point j and the operator $e^{\delta t \mathbb{W}_s}$ with the counting field s (the strength of tilting), we approximate the next-step probability vector $|\hat{Q}_j^{\theta_j}\rangle = e^{\delta t \mathbb{W}_s}|\hat{P}_j^{\theta_j}\rangle / Z_{j+1}(s)$ with the normalization constant $Z_{j+1}(s)$. We learn the evolved distribution with a variational ansatz $\hat{P}_{j+1}^{\theta_{j+1}}(\mathbf{x})$ by minimizing the loss function of its distance to the evolved distribution. We consider the Kullback-Leibler (KL)-divergence at each time point j:

$$\begin{aligned} D_{KL}[\hat{P}_{j+1}^{\theta_{j+1}}||\hat{Q}_j^{\theta_j}] &= \sum_{\mathbf{x}} \hat{P}_{j+1}^{\theta_{j+1}}(\mathbf{x})[\ln \hat{P}_{j+1}^{\theta_{j+1}}(\mathbf{x}) - \ln \hat{Q}_j^{\theta_j}(\mathbf{x})] \\ &= \sum_{\mathbf{x}} \hat{P}_{j+1}^{\theta_{j+1}}(\mathbf{x})[\ln \hat{P}_{j+1}^{\theta_{j+1}}(\mathbf{x}) - \ln e^{\delta t \mathbb{W}_s} \hat{P}_j^{\theta_j}(\mathbf{x})] + \ln Z_{j+1}(s), \end{aligned} \tag{S1}$$

where D_{KL} denotes the KL-divergence and the summation $\sum_{\mathbf{x}}$ is over all the possible configurations.

The probability distributions $\hat{P}_{j+1}^{\theta_{j+1}}(\mathbf{x}), \hat{P}_j^{\theta_j}(\mathbf{x})$, with the parameters denoted by θ, can be parameterized by the VAN. Under the tilted dynamical operator, the probability vector acted on by the operator $e^{\delta t \mathbb{W}_s}|\hat{P}_j^{\theta_j}\rangle$ is not normalized, i.e., the normalization constant $Z_{j+1}(s)$ is unknown in prior and needs to be estimated. Importantly, the VAN represents an automatically normalized model by design [2], and thus the distribution $\hat{P}_{j+1}^{\theta_{j+1}}(\mathbf{x})$ is renormalized after each iteration. With the renormalization, we can draw samples from the normalized distribution $\hat{P}_{j+1}^{\theta_{j+1}}(\mathbf{x})$ to evaluate the loss function.

The loss function at each time step is defined as the KL-divergence without the normalization constant:

$$\begin{aligned} \mathcal{L}_{j+1} &= \sum_{\mathbf{x}} \hat{P}_{j+1}^{\theta_{j+1}}(\mathbf{x})[\ln \hat{P}_{j+1}^{\theta_{j+1}}(\mathbf{x}) - \ln \mathbb{T}_s \hat{P}_j^{\theta_j}(\mathbf{x})] \\ &= \mathbb{E}_{\mathbf{x} \sim \hat{P}_{j+1}^{\theta_{j+1}}}[\ln \hat{P}_{j+1}^{\theta_{j+1}}(\mathbf{x}) - \ln \mathbb{T}_s \hat{P}_j^{\theta_j}(\mathbf{x})], \end{aligned} \tag{S2}$$

where the transition operator $\mathbb{T}_s = (\mathbb{I} + \delta t \mathbb{W}_s) \approx e^{\delta t \mathbb{W}_s}$ for small δt. In the expectation, $\mathbf{x}$ are samples drawn from the distribution $\hat{P}_{j+1}^{\theta_{j+1}}(\mathbf{x})$: $\mathbb{E}_{\mathbf{x} \sim \hat{P}_{j+1}^{\theta_{j+1}}}[\dots] \approx \sum_{\mathbf{x}} \hat{P}_{j+1}^{\theta_{j+1}}(\mathbf{x})[\dots]$. In the main text, we use the notation $\sum_{\mathbf{x}} \hat{P}_{j+1}^{\theta_{j+1}}(\mathbf{x})[\dots]$ for the summation of the samples generated from the distribution.

Due to the nonnegativity of the KL-divergence in Eq. (S1) we have:

$$\mathcal{L}_{j+1} \geq -\ln Z_{j+1}(s), \tag{S3}$$

where the equality holds when the VAN accurately learns the evolved probability distribution. Then, the minimized loss leads to the normalization constant at each time step, with more details provided below.

The operator $\mathbb{W}_s$ can be specified for each model. For KCMs, where the state transition occurs for states with only one-spin difference, we only need to evaluate N (the number of spin sites) manipulations to have all the transitions into or out from each sampled state for $\mathbb{T}_s \hat{P}_j^{\theta_j}(\mathbf{x})$. This procedure can be executed in parallel for all the samples in the batch.

The parameters θ_{j+1} can be updated by minimizing the loss function [2]:

$$\begin{aligned} \nabla_{\theta_{j+1}} \mathcal{L}_{j+1} &= \nabla_{\theta_{j+1}} \sum_{\mathbf{x}} \hat{P}_{j+1}^{\theta_{j+1}}(\mathbf{x})[\ln \hat{P}_{j+1}^{\theta_{j+1}}(\mathbf{x}) - \ln \mathbb{T}_s \hat{P}_j^{\theta_j}(\mathbf{x})] \\ &= \sum_{\mathbf{x}} \{[\nabla_{\theta_{j+1}} \hat{P}_{j+1}^{\theta_{j+1}}(\mathbf{x})] \cdot [\ln \hat{P}_{j+1}^{\theta_{j+1}}(\mathbf{x}) - \ln \mathbb{T}_s \hat{P}_j^{\theta_j}(\mathbf{x})] + \hat{P}_{j+1}^{\theta_{j+1}}(\mathbf{x}) \nabla_{\theta_{j+1}} \ln \hat{P}_{j+1}^{\theta_{j+1}}(\mathbf{x})\} \\ &= \mathbb{E}_{\mathbf{x} \sim \hat{P}_{j+1}^{\theta_{j+1}}} \{[\nabla_{\theta_{j+1}} \ln \hat{P}_{j+1}^{\theta_{j+1}}(\mathbf{x})] \cdot [\ln \hat{P}_{j+1}^{\theta_{j+1}}(\mathbf{x}) - \ln \mathbb{T}_s \hat{P}_j^{\theta_j}(\mathbf{x})]\}, \end{aligned} \tag{S4}$$

where we have used the log-derivative technique $\nabla_\theta P_\theta = P_\theta \cdot \nabla_\theta \ln P_\theta$ and relation $\mathbb{E}_{\mathbf{x}\sim \hat{P}^{\theta_{j+1}}_{j+1}}[\nabla_{\theta_{j+1}} \ln \hat{P}^{\theta_{j+1}}_{j+1}(\mathbf{x})] = \nabla_{\theta_{j+1}} \sum_{\mathbf{x}} \hat{P}^{\theta_{j+1}}_{j+1}(\mathbf{x}) = \nabla_{\theta_{j+1}} 1 = 0$ when the number of samples is sufficient. The variance reduction is included to reduce the variance of the loss function [2].

This gradient estimator is called REINFORCE [3] in the machine learning community. As a different way of updating the parameters, a time-dependent variational method was developed in [4, 5] and can be used for training the neural network. However, inverting the Fisher-metric matrix may be computationally demanding when the number of neural-network parameters increases.

II. EVALUATING THE DYNAMICAL PARTITION FUNCTION

Although the VAN was applied to evolve quantum systems [4, 6], there is a lack of a framework to track the unnormalized probability evolution under tilted dynamics, which is necessary to uncover the phase transition. Here, we fill this gap. With the learnt VAN, we can evaluate the dynamical partition function under the tilted operator and reveal the phase transition. When learning VAN at each time step, the normalization factor as the volume change of the distribution has the information of the partition function. We record the normalization factor at each time step and multiply all of them to obtain the dynamical partition function.

Specifically, the dynamical partition function is obtained by applying $e^{t\mathbb{W}_s}$ to the steady state:

$$Z_t(s) = \langle -|e^{t\mathbb{W}_s}|\mathrm{ss}\rangle, \tag{S5}$$

where $|\mathrm{ss}\rangle$ is the steady state vector and $\langle -| = \sum_{\mathbf{x}}\langle \mathbf{x}|$ is the flat state as the left eigenvector of the generator. For the system with detailed balance, the Hermitian form of the operator can be used by a similarity transformation $\mathbb{W}_s \to \mathbb{P}^{-1/2}\mathbb{W}_s\mathbb{P}^{1/2}$, where $\mathbb{P}^{-1/2}$ is a diagonal matrix of the steady-state probability vector [7].

Evaluating $Z_t(s)$ by Eq. (S5) requires full matrix manipulation. Alternatively, we can obtain the normalization constant by using samples from the learnt VAN as follows. By the Suzuki-Trotter decomposition [1], the partition function Eq. (S5) can be evaluated sequentially with J total time steps:

$$\begin{aligned} Z_t(s) &\approx \langle -|[e^{\delta t\mathbb{W}_s}]^J|\mathrm{ss}\rangle \\ &= \langle -|[e^{\delta t\mathbb{W}_s}]^{J-1}e^{\delta t\mathbb{W}_s}|\mathrm{ss}\rangle \\ &\approx \langle -|[e^{\delta t\mathbb{W}_s}]^{J-1}|\hat{P}^{\theta_1}_1\rangle Z_1(s), \\ &\approx \dots \\ &\approx \langle -|\hat{P}^{\theta_J}_J\rangle \prod_{j=1}^{J} Z_j(s) \\ &= \prod_{j=1}^{J} Z_j(s), \end{aligned} \tag{S6}$$

where the probability vector at each time step, such as $|\hat{P}^{\theta_1}_1\rangle$, is learnt by the VAN as a normalized distribution. The first approximation has an error incurred by the Suzuki-Trotter decomposition, and the remaining approximations have the error resulting from training the VAN. The last step is due to the normalization property of the VAN.

The normalization constants $Z_j(s)$ can be estimated by the variational free energy $\mathcal{F}^{\theta_j}_j(s)$ when the VAN learns the distribution. That is,

$$\ln Z_t(s) \approx \sum_{j=1}^{J} \ln Z_j(s) = -\sum_{j=1}^{J} \mathcal{F}_j(s) \geq -\sum_{j=1}^{J} \mathcal{F}^{\theta_j}_j(s), \tag{S7}$$

where the equality in the last step holds when the VAN accurately learns the evolved distribution at each time step. We remark that since we do not have the energy function (Hamiltonian) from the model, it is infeasible to calculate the normalization constant by Eq. (S12) in [2]. Note that the distribution $\hat{P}^{\theta_j}_j$ yielded by the VAN cannot be directly used to calculate the partition function either because the VAN is already normalized.

The variational free energy at the time step $j+1$ is estimated from the loss function Eq. (S2) at the end of training:

$$\mathcal{F}^{\theta_{j+1}}_{j+1}(s) = \mathcal{L}_{j+1}. \tag{S8}$$

To better approximate the summation on all the configurations in Eq. (S2) for calculating $\mathcal{L}_{j+1}$, one may consider reweighting the expectation [8] of calculating the variational free energy. For example, $\mathbb{E}_{\mathbf{x}\sim\hat{P}^{\theta_{j+1}}_{j+1}}[\dots] = \sum_{\mathbf{x}\sim\hat{P}^{\theta_{j+1}}_{j+1}}\{\hat{P}^{\theta_{j+1}}_{j+1}(\mathbf{x})\cdot[\dots]/[\sum_{\mathbf{x}'\sim\hat{P}^{\theta_{j+1}}_{j+1}}\hat{P}^{\theta_{j+1}}_{j+1}(\mathbf{x}')]\}$, and the summations are on all the drawn samples. This reweighting is the same as the importance sampling, Eq. (9) in [8], which can help the accuracy of the sampled average. We find this reweighting procedure useful in examples.

The moments of the dynamical observable are obtained by taking derivatives of the dynamical partition function. For example, the average dynamical activity per unit time and site follows from the partition function:

$$k_t(s) = -\frac{1}{Nt}\frac{d}{ds}\ln Z_t(s). \tag{S9}$$

Higher moments can be estimated similarly.

A. The method of projection

In examples, we evolve the system for a sufficiently long time when the accumulation rate of the partition function converges. A longer simulation time leads to a state closer to the steady state. We now provide another method to save the computational time of evolving the state. It employs the convergence of the dynamical partition function in the long-time limit to extrapolate from finite time.

In the long-time limit, Eq. (S5) becomes $Z_t(s) \sim e^{t\theta(s)}$, where $\theta(s)$ is the scaled cumulant generating function (SCGF) [9]. One can use the variational Monte-Carlo to calculate the SCGF $\theta(s)$ in the long-time limit [10]: Note that the VAN there represents the wave function with its square being normalized, but here it represents the probability distribution.

With the estimated SCGF $\theta(s)$, we rewrite the dynamical partition function as:

$$Z_t(s) = e^{t\theta(s)}\langle -|e^{t[\mathbb{W}_s-\theta(s)]}|\mathrm{ss}\rangle. \tag{S10}$$

Correspondingly, the modified tilted generator is:

$$\hat{\mathbb{W}}_s = \sum_{\mathbf{x},\mathbf{x}'\neq\mathbf{x}} e^{-s}w_{\mathbf{x},\mathbf{x}'}|\mathbf{x}'\rangle\langle\mathbf{x}| - \sum_{\mathbf{x}}[r_{\mathbf{x}}+\theta(s)]|\mathbf{x}\rangle\langle\mathbf{x}|. \tag{S11}$$

The operator $\hat{\mathbb{W}}_s$ has the zero largest eigenvalue, but it is not a stochastic operator. Indeed, the operator $\hat{\mathbb{W}}_s$ is different from the Doob operator [11], as the latter can be annihilated by the left flat state.

In the long-time limit, the logarithmic dynamical partition function eventually converges to a linear function of time with the slope θ, and all but the leading eigenvector are exponentially dampened. Therefore, the slope of $\ln\langle -|e^{t[\mathbb{W}_s-\theta(s)]}|\mathrm{ss}\rangle$ should converge to nearly zero, at which the simulation can be stopped to extrapolate the long-time limit by using the SCGF. Around the phase transition, the time scale of the convergence may diverge.

III. ENHANCEMENT ON TRACKING THE PHASE TRANSITION

The accuracy of the VAN is especially demanding near the phase transition because configurations at different phases need to be sampled to train the VAN and the configurations can be rare. To improve the efficiency, we provide two methods to help sample rare configurations: importance sampling and proper initialization for the VAN. These strategies may be employed near the phase transition of KCMs.

A. Importance sampling

For KCMs, the active phase has many spin flips, so the number of up spins should not be small. There are many such configurations for the active phase. For the inactive phase, spins do not flip often, and the number of up spins must be small, which are rare configurations. These rare configurations are hard to sample, leading to a challenge of training the VAN from the active to the inactive phase.

To sample rare configurations more effectively, we leverage importance sampling. We first employ a distribution where rare configurations have higher probability. We sample from this distribution and perform probability reweighting when calculating the expected value over the VAN distribution:

$$\mathbb{E}_{\mathbf{x}\sim\hat{P}^{\theta}}\{\dots\} = \mathbb{E}_{\mathbf{x}\sim\tilde{P}}\{(\hat{P}^{\theta}/\tilde{P})\dots\} = \mathbb{E}_{\mathbf{x}\sim\tilde{P}}\{\exp(\ln\hat{P}^{\theta}-\ln\tilde{P})\dots\}, \tag{S12}$$

where $\hat{P}^\theta$ is the distribution by the VAN, and $\tilde{P}$ is a prior distribution.

For KCMs, we use the binomial distribution as $\tilde{P}$ with the coefficient of the binomial distribution $p = 1/N$, where N is the total number of spins, such that the average up spin from the distribution is 1. Therefore, the configurations with only one up spin can be sampled out frequently. In general, importance sampling needs to be designed for the specific problem, with the distribution $\tilde{P}$ tailored to sample particular configurations. After adding the samples from the importance samples, the learning rate may be decreased to reduce the loss variance.

With this strategy, the estimation on the SCGF at the steady state and the finite-time evolution becomes more accurate for all values of the counting field s. The detailed setting of the importance sampling may differ between these two tasks. For calculating the SCGF, adding approximately fifty samples from the important sampling to the one thousand batch ($\sim 5\%$) from the VAN improved the efficiency of estimating the SCGF. To track the finite-time evolution, we added a few samples, e.g., one sample, from the important sampling to the batch with a thousand samples from the VAN. This accelerated the search of the inactive phases of KCMs. The proper number of added samples is dependent on the system size and specific problems and may need to be fine-tuned to control the system in certain phases. Under the same number of samples from the importance sampling, the PixelCNN is more robust under various s, that is, the inactive and active phases were both efficiently learnt. The RNN with importance sampling is more sensitive to the counting field s.

The active importance sampling for neural networks was proposed in [12], where umbrella sampling and replica exchange were used. However, it is not directly applicable in the present setting because the normalization constant of the windowing functions in their Eq. (18) is not computable for the current case. Instead, here we employed a normalized prior distribution, compatible with the rare configurations of KCMs.

B. Proper choices on the initialization of the VAN

To accurately estimate the SCGF at the steady state with $s > 0$, we conducted the variational Monte Carlo simulation [10], both from small to large s and from large to small s. When going from small to large, we used the VAN trained under small s as the initial VAN for the next larger one, and vice versa when going from large to small. This estimation employs the VAN either from the active phase with smaller s or the inactive phase with larger s, enabling us to find the more proper phases for the current s. Then, we took the larger value of the SCGF from the two results, serving as the best estimation on the SCGF.

For finite time, one may save the VAN at each time point and various s. Then, it is useful to employ information from different s by using the VAN under the neighbor values of the chosen s to calculate the evolved distribution. This helps to improve the efficiency of sampling rare configurations at different phases, which generates a more accurate estimation of the dependence on s. This strategy shares a similar spirit of the replica exchange in [13], where two trajectories corresponding to different s are swapped. Here, configurations at each time point rather than trajectories were shared between different s.

To capture the time point where the phase transition occurs, we can also employ the method of choosing a proper initialization of the VAN at each time point. To better capture the phase transition, two initialized VANs may be used: one from the previous time point, and the other from the steady-state variational Monte-Carlo calculation. After training, the one with lower loss is chosen, such that the model closer to the optimal solution is found at any time point.

Based on the above two strategies of importance sampling and proper VAN initialization, we have a protocol for uncovering the full phase diagram of dynamical observables for a given nonequilibrium system. Taking the active-inactive phase transition of the KCM as an example, one can first calculate the SCGF at the steady state by using the variational Monte-Carlo method, with the importance sampling or a proper initialization of the VAN, such that the active-inactive phase transition versus the counting field can be precisely revealed. Second, the present method tracks time evolution of the dynamical observable. If there is a mismatch between the long-time dynamical observable and that from the SCGF, such as around the phase transition point, one may turn on the importance sampling at a certain time point to better capture the active-inactive phase transition over time.

IV. VOTER MODEL IN TWO DIMENSION

We consider the voter model describing consensus formation in a lattice of spins [14]. Since it has an advantage of being analytically solvable, it serves as an example to validate our method. In this model, each configuration $|\mathbf{x}\rangle \equiv (x_1, x_2, \ldots, x_N)$ has the binary spins $x_i = 0, 1$ $(i = 1, \ldots, N)$, corresponding to the up and down states. During the time evolution, each spin updates its "opinion" to its nearest neighbors.

We study the system on $2D$ lattice with $N = L^2$. Its dynamics can be described by Eq. (1) with the following transition rate from $|\mathbf{x}\rangle$ to $|\mathbf{x}^{'}\rangle$: if $|\mathbf{x}\rangle$ to $|\mathbf{x}^{'}\rangle$ has only one spin difference at the i-th site,

$$w_{\mathbf{x},\mathbf{x}'} = \sum_{x' \in f_i(|\mathbf{x}\rangle)} |x_i - x_{i'}|/2^L, \tag{S13}$$

where $f_i(|\mathbf{x}\rangle)$ denotes all the nearest neighbors of the i-th site in the configuration $|\mathbf{x}\rangle$. Thus, the spin flips according to the proportion of the opposite state at the nearest neighbors: it will not flip if all its nearest neighbors have the same state, and will have a flipping rate 1 if all the nearest neighbors have the opposite state. An illustration is given in Supplementary Fig. 1a.

We apply the VAN to track the probability distribution of the system. Specifically, we start from a uniform initial distribution of the spin configurations and evolve the system sufficiently long time with observing the convergence. We consider boundary conditions with randomly assigned $1, 0$ to the off-boundary sites [14], such that the boundary sites are subject to a similar rule of time evolution. We then estimate the statistics from the joint distribution to evaluate our method.

For the average density, it has been shown that the system in finite size eventually reaches consensus, i.e., with all spins in the same state as the only absorbing configuration of the system. The amount of all-up and all-down configurations depends on the initial ratio of the up and down spins [14]. Since we start from a uniform distribution of the configurations, the evolved distribution should have half all-up and half all-down configurations, giving a converged average density $\langle n(t)\rangle = 0.5$. From the VAN method, the average density of up spins $\langle n(t)\rangle$ does converge to the stable value 0.5, matching with this analysis (Supplementary Fig. 1b).

We further consider the two-sites correlation function $G(x_i, x_{i'}) = \langle |x_i - x_{i'}|\rangle$, where the average is on the drawn configurations. A characteristic quantity related to the correlation function is the interfacial density, which accounts for the fraction ρ of neighboring voters with opposite opinions. It is estimated from the correlation function: $\rho = [1 + G(x_i, x_{i'})]/2$ for the nearest neighbors $x_i, x_{i'}$ with distance 1 on the lattice. According to Eq. (8.27) in [14], the analytical result for the time dependence of the interfacial density $\rho(t) \sim \pi/(2\ln t)$, which is valid only in the long-time limit. Based on the VAN, the estimated $\rho(t)$ matches with the analytic result in the long-time limit (Supplementary Fig. 1c).

V. KINETICALLY CONSTRAINED MODELS

We next provide details for kinetically constrained models, in 1D, 2D and 3D separately.

A. One-dimensional cases

For 1D models, we chose the boundary sites with up spins [15]. The results are plotted in Supplementary Fig. 2 with positive s and Supplementary Fig. 3 with negative s. They show consistent behaviors with the previous results [15–17], validating our approach for one-dimensional systems.

B. Two-dimensional cases

We set the boundary sites with down spins to be consistent with the literature [10]. Since all the boundary sites have down spins, the configuration with all down spins is an absorbing configuration. Thus, it should be excluded from the system evolution because otherwise it will absorb the probabilities of all other configurations. For the 2D SE model, the first spin is fixed up to overcome this issue. For the 2D FA model, we used the setting without fixing the first spin up and excluded the disconnected configuration with all down spins, consistent with the setting in [10]. Specifically, in the last spin site, we manually chose it to be up if every previous site has down spins by setting the probability of the last up spin to be 1, such that the configuration with all down spins has probability zero. This setting ensures the normalization condition of the total probabilities.

We take the 2D SE model as an example to demonstrate the quantification on the accuracy of the training. To evaluate the accuracy, there are two approaches: (1) comparing with other methods, and (2) using an "intrinsic" estimator such as the loss function of the training process. For (1), we have compared the evolving distribution from the VAN with the numerically exact result for small system sizes (Supplementary Fig. 4a). The small KL-divergence demonstrates that the training does not suffer from a serious error accumulation over time. The KL-divergence mainly

increases when the system transits from the active phase to the inactive phase, where the error can be further reduced by using more training epochs near the transition time or employing the strategies in Supplementary Sect. III.

For larger system sizes, such as $L = 5, 6$ in 2D, no other methods are available. Thus, the only currently available way to evaluate the accuracy is by the loss function Eq. (S2) based on the KL-divergence. In general, the lower value of the loss function implies more accurate training, with the lower bound in Eq. (S3). Note that the loss function here is not the KL-divergence divergence of two probability distributions, because the probability is no longer normalized under the tilted generator. The loss function does not reach zero, even when the VAN is faithfully learnt, but reaches the lower bound by the dynamical partition function.

To evaluate how the training accuracy depend on the number of epochs under various system sizes, we consider the counting field with the phase transition, use 100 epoch at each time point (except for the first time point), and examine whether the loss function increases with a smaller number of training epochs. The result shows that smaller epoch gives larger loss (Supplementary Fig. 4b) and the loss increases with the system size (Supplementary Fig. 4c). For system sizes larger than the chosen ones, it may require > 100 epoch to have the loss sufficiently small. Then, according to Supplementary Table 1, the computational time for each counting field s will be the order of $\mathcal{O}(10^2)$ hours.

For the other 2D and 3D models, the active-inactive phase transition is also accurately tracked (Supplementary Fig. 5), with the relative error for the small lattice size shown in the inset. Since the computational time required is long for tracking time evolution, the chosen lattice size L is $L = 4$ for the 2D quantum system [2]. Here, we need to further estimate time evolution under various values of the counting field s and thus considered $L = 4, 5, 6$ in 2D and $L = 2, 3, 4$ in 3D.

For $s > 0$ in 2D, we used values of the counting field logarithmically, i.e., $\log_{10} s \in [-2, 0]$ with the interval 0.1. With the result under discrete values of s, we used the interpolation and smoothed the phase transition line, which does not affect the physical result on the phase transition, such as its scaling on time. The present finite-time result at the last time point also converges to the SCGF at the steady state (Supplementary Fig. 6), calculated from the variational Monte-Carlo method [10]. The remaining deviation comes from the fact that the last time point may have not fully reached the steady state and the error from the Suzuki-Trotter decomposition.

For $s < 0$ in 2D, the values of the counting field were also chosen logarithmically, i.e., $\log_{10}(-s) \in [-3, 0]$ with the interval 0.2. We use $\nu = 1 - e^s$ as the horizontal axis. Here, we have fixed the left-up spin up for both the 2D FA and 2D SE models to better compare their characteristic spatial structures. Using more samples increases the accuracy of estimating the average density, and a longer simulation time may be required to reach closer to the steady state.

C. Three-dimensional cases

We consider the South-East-Front (SEF) model in 3D as a natural extension of the South-East model in 2D. The SEF model in 3D has f_i count the number of up spins only for the left, above, and back nearest neighbors. To implement the VAN, we used a three-dimensional version of MADE [18] by first translating the three-dimensional lattice into a one-dimensional chain.

We applied the method to track the active-inactive phase transition of the 3D SEF model with $L = 2, 3, 4$ (Fig. 2). The critical exponent of size $\alpha \gtrsim 1$, which is similar to the 1D [17] and 2D [10] cases. The critical exponent of time $\beta \approx 1$ is similar to the 1D case in [15] and the 2D case obtained in the present work. The results of the 2D and 3D models demonstrate that our approach is able to track the finite-time evolution for higher dimensional systems of nonequilibrium statistical mechanics.

D. Computational details for kinetically constrained models

We list the number of depths and widths of the neural network with the best performance in our attempts for each kinetically constrained models under consideration (Supplementary Table 1). The table also provides the corresponding computational time on various system sizes and dimensions.

The choice of depth and width depends on the architecture of the VAN, e.g., RNN, gated PixelCNN, MADE. As recorded in Supplementary Table 1, we have chosen depth 1 for RNN, and deeper layers for the gated PixelCNN and MADE. These choices are based on both the accuracy and the computational time. First, the accuracy of the VAN depends on the depth and width of the neural network. The RNN with depth 1 has the relative error smaller than 10^{-3} (Supplementary Fig. 2), which is similar to those of the gated PixelCNN and MADE with deeper layers. For the width of RNN, we have used $8, 16, 32, 64, 128$, and found that $64, 128$ generally give lower loss for the one-layer RNN. The width of the gated PixelCNN and MADE is chosen under a same procedure. Second, for the computational time, deeper and wider neural networks require longer time. Especially, the RNN has a special architecture compared

with the gated PixelCNN and MADE: the RNN cell passes both the variable and hidden state at each site of the lattice sequentially (Methods). Such an architecture of the RNN causes longer computational time than the gated PixelCNN and MADE (Supplementary Table 1), and the computational time will be further increased with depth, i.e., the number of layers for the hidden states. Thus, the RNN with depth 1 is typically used [10]. A larger number of hidden states and more hidden layers can be employed to reach a lower error, with the cost of longer computational time.

Furthermore, we generated configurations with symmetry of the lattice systems, e.g., the parity symmetry for 1D spin systems and the rotational plus reflectional symmetry for 2D cases. The imposed symmetry does not significantly improve the accuracy of the training or the estimation on the dynamical partition function in the KCMs, and requires longer computational time. Therefore, symmetry was not included in most of our numerical implementations but was added as an option in our code package for specific tasks that require the imposing of symmetry.

VI. SUPPLEMENTARY FIGURES

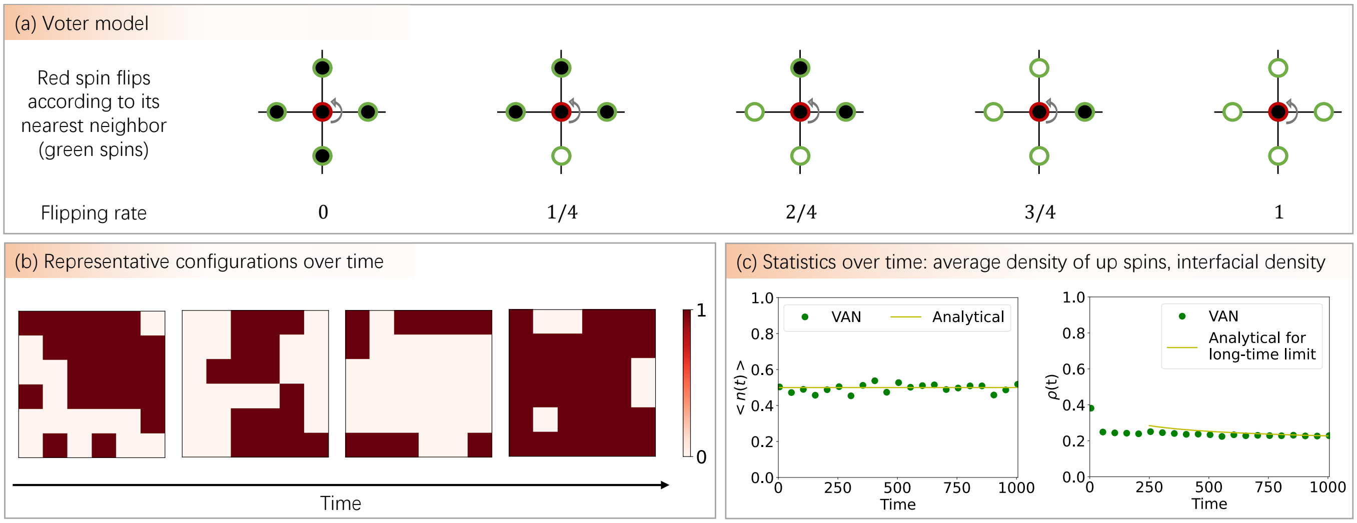

Supplementary Figure 1. Results for the voter model. (a) A schematic on the role of spin flips in the voter model. The filled (unfilled) circle is up (down) spin. (b) The configurations tend to reach consensus over time, with more spins in the same state (0 or 1). The color denotes the up ($x_i = 1$) or down ($x_i = 0$) spins. (c) The VAN gives time evolution of the statistics, including the average density of up spins $\langle n(t) \rangle$ and the interfacial density $\rho(t)$ over time. The $\rho(t)$ matches with the analytical solution available only for the long-time limit. Parameters: the lattice size is $L = 6$; the VAN is based on the RNN with depth 1 and width 16; the training has 100 samples in each batch, 3000 epoch at the first time step, 30 epoch at the following time steps; the time evolution has $\delta t = 0.05$, and $2 * 10^4$ time steps. The samples for estimating the statistics are drawn from the last two epochs after convergence of the training.

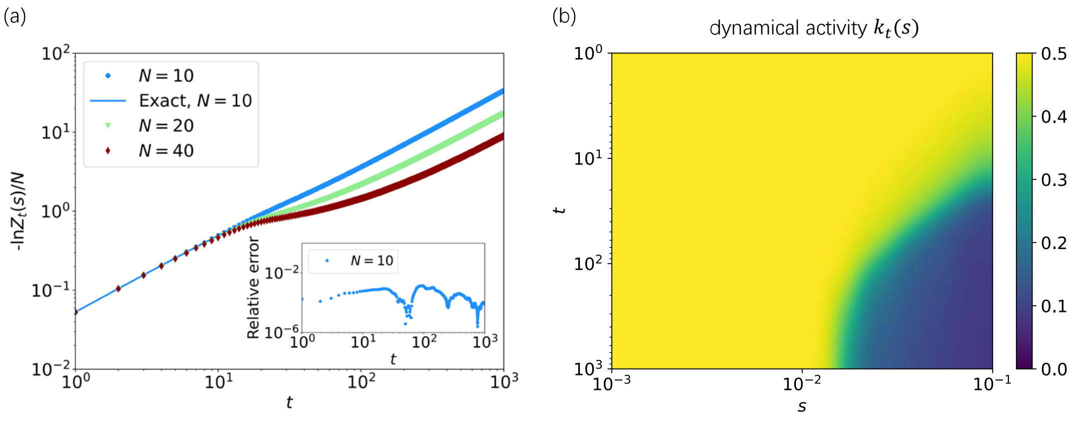

Supplementary Figure 2. The dynamical partition function and phase transition for the 1D FA model. (a) The logarithmic dynamical partition function $\ln Z_t(s)$ over time ($c = 0.5, s = 0.1, N = 10, 20, 40$). $N = 10$ is compared with the numerically exact result by storing the full probability distribution. The time step is set as $\delta t = 0.1$, and the plotted time points are taken from every 10 steps to better visualize the comparison with the numerically exact result for $N = 10$. (b) The dynamical activity $k_t(s)$ denoted by the color reveals the active-inactive phase transition over time for the 1D FA with $N = 20$ and $c = 0.5$.

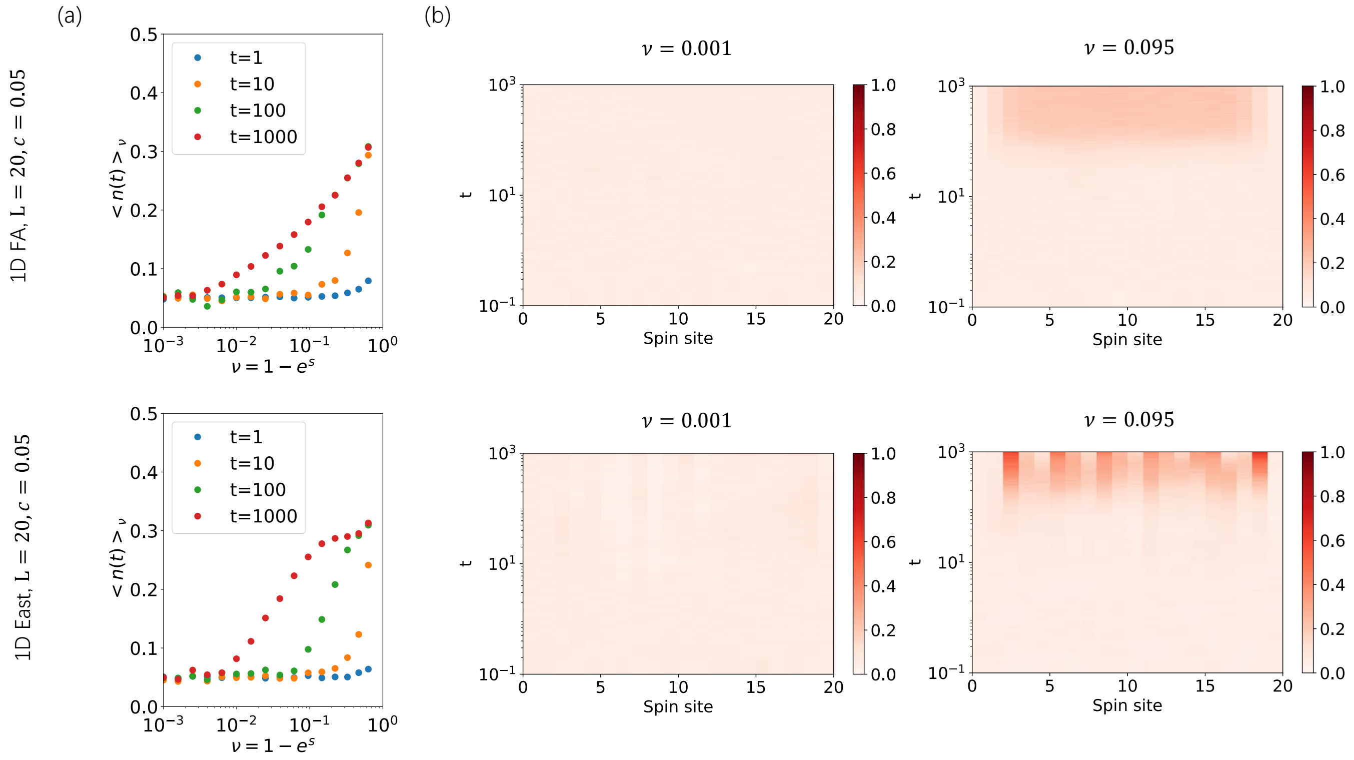

Supplementary Figure 3. Emergence of active phases for 1D models. The 1D FA (top) and East (bottom) models with $c = 0.05$ and negative s. (a) The average up spins for different negative s versus $\nu = 1 - e^s$ at several time points. (b) For $c = 0.05$, the East model forms structures with up spins separated by down spins. The FA model does not have such structures but more up spins over time at the middle of the 1D chain. The color denotes the average number of up spins. These behaviors are consistent with the previous result by the tensor network [15].

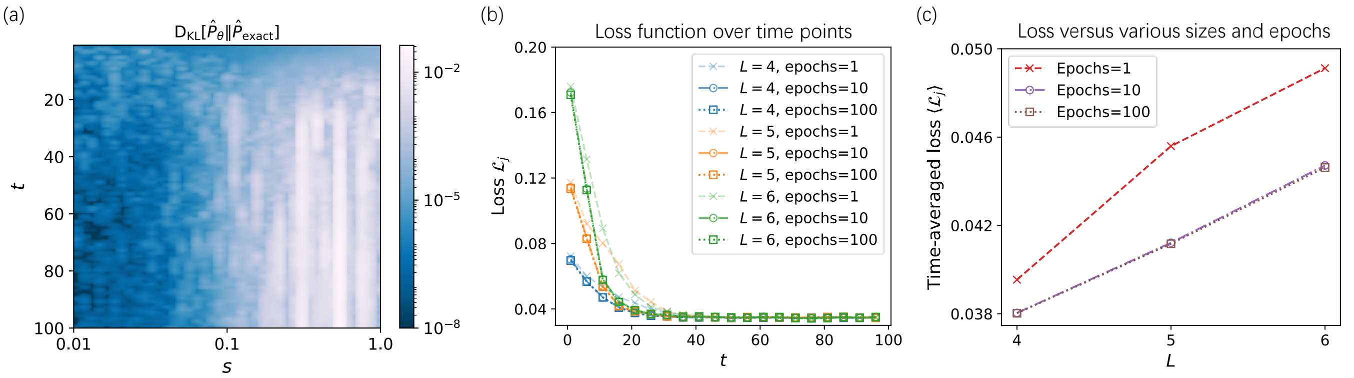

Supplementary Figure 4. The training accuracy and its dependence on the system size. The analysis is based on the 2D SE model as an example. (a) The KL-divergence between the distribution $\hat{P}_\theta$ from the VAN and the numerically exact $\hat{P}_{\text{exact}}$ for $L = 4$. The KL-divergence increases near the region of the active-inactive dynamical phase transition, suggesting to use more training epochs in this critical region or employ strategies in Supplementary Sect. III. (b) The loss function, Eq. (S2), for different system sizes $L = 4, 5, 6$ under $\log_{10} s = -0.6$ with the phase transition. We choose $\delta t = 0.05$ and show the result at $t = 1, 6, 11, \ldots$, with the total evolution time $t = 100$. We utilize 100 training epochs at each time point (except for the first time step), and show the loss function when the training reaches 1, 10, and 100 epoch respectively. (c) The time-averaged loss value in (b) from $t = 1$ to 100 for different training epochs and system sizes.

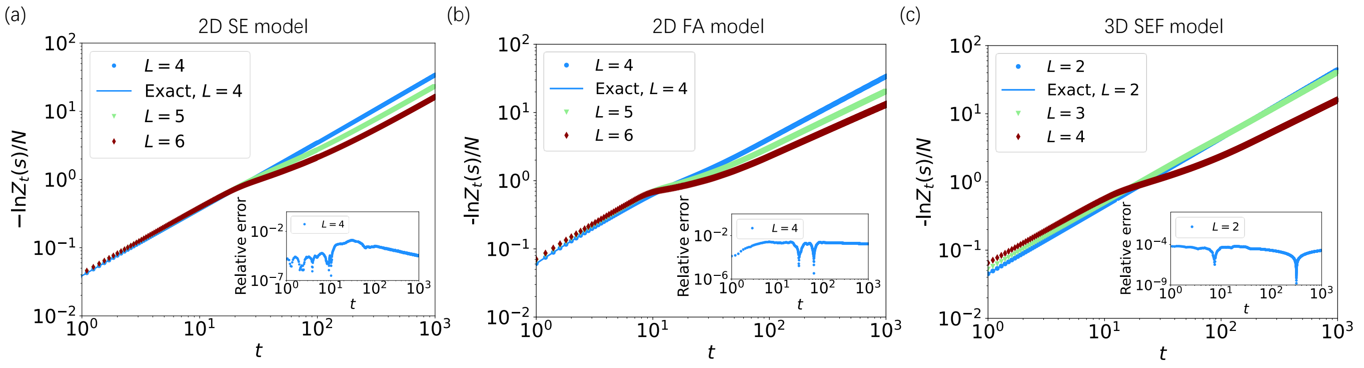

Supplementary Figure 5. Tracking time evolution of the dynamical partition function for KCMs. The systems are the 2D SE, 2D FA and 3D SEF models. (a) The logarithmic dynamical partition function $\ln Z_t(s)$ over time in a $L \times L$ lattice ($c = 0.5, s = 0.1, L = 4, 5, 6$). The tilt of the curve indicates the active-inactive phase transition (see text). $L = 4$ is compared with the numerically exact solution by storing the full probability distribution, and the relative error is plotted in the inset. (b) The same as (a) for the 2D SE model with $c = 0.5, s = 0.1, L = 4, 5, 6$. (c) The same as (a) for the 3D SEF model with $c = 0.5, s = 0.1, L = 2, 3, 4$.

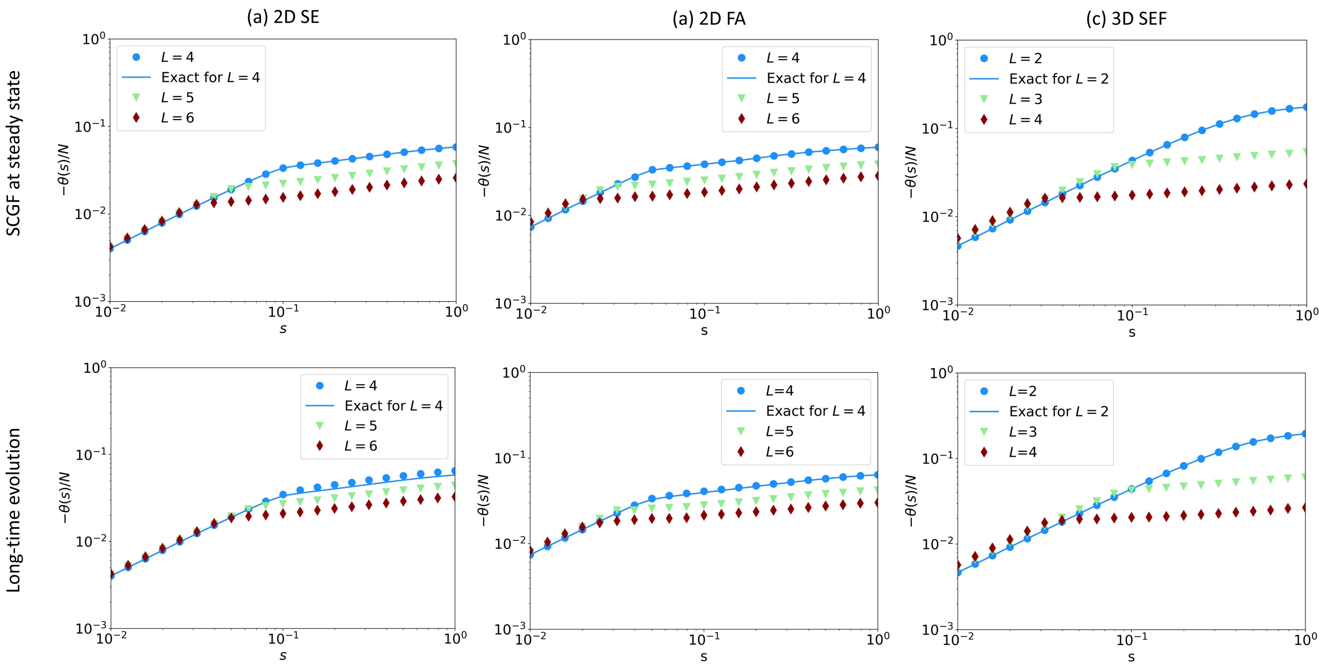

Supplementary Figure 6. The consistency between the long-time evolution and SCGF at the steady state for (a) the 2D SE, (b) the 2D FA, and (c) the 3D SEF models. (Top) The SCGF at the steady state calculated from the variational Monte Carlo method. (Bottom) The logarithm of the dynamical partition function at the last time point, which agrees with the top panel. For the smallest lattice size in each panel, the present method matches well with the numerically exact result (line).

Supplementary Table 1. The table for the chosen VAN size and the computational time for the time evolution and the steady-state calculation. The computational time is under one value of the counting filed s. The systems under 1D, 2D, 3D, and with different lattice lengths are considered. For the case of the time evolution, the lattice size with + means that the computational time includes that of calculating the numerically exact result, which are approximately 0.00011 hour for 1D, 0.00005 hour for 2D, and 0.00290 hour for 3D under the corresponding number of time steps. A certain number of time steps is chosen in various dimensions. For the steady-state calculation from the variational Monte-Carlo method, the computational time of the 2D case is listed here as in [10]: the result is compared with the present method under the long-time limit (Supplementary Fig. 6). All the computational time is based on a single core GPU ($\sim 25\%$ usage) of Tesla-V100. The learning rate is 10^{-3} with the Adam optimizer [19], and the batch size is 1000.

Type	Finite time: the first time step uses the order of 10^3 epochs, and the following time steps use 100 epochs.			Steady state by variational Monte-Carlo, 5000 epochs
Dimension	1	2	3	2
Time steps	10^4	4×10^3	4×10^3	NaN
VAN type	RNN	Gated PixelCNN (RNN)	MADE	Gated PixelCNN
VAN size (depth, width)	1, 128	3, 32 (1, 128)	4, 16	3, 32
Lattice size L	$10^*/20/40$	$4^*/5/6$	$2^*/3/4$	4/5/6
Computational time for one s value (hour)	16.83/11.37/65.28	4.09/8.01/12.24 (16.63/32.12/56.43)	2.68/5.74/17.64	0.06/0.09/0.24

[1] M. Suzuki, Commun. Math. Phys. **51**, 183 (1976).
[2] D. Wu, L. Wang, and P. Zhang, Phys. Rev. Lett. **122**, 080602 (2019).
[3] R. J. Williams, Machine Learning **8**, 229 (1992).
[4] M. Reh, M. Schmitt, and M. Gärttner, Phys. Rev. Lett. **127**, 230501 (2021).
[5] M. Reh and M. Gärttner, Mach. Learn. Sci. Technol. **3**, 04LT02 (2022).
[6] D. Luo, Z. Chen, J. Carrasquilla, and B. K. Clark, Phys. Rev. Lett. **128**, 090501 (2022).
[7] J. P. Garrahan, R. L. Jack, V. Lecomte, E. Pitard, K. van Duijvendijk, and F. van Wijland, J. Phys. A **42**, 075007 (2009).
[8] K. A. Nicoli, S. Nakajima, N. Strodthoff, W. Samek, K.-R. Müller, and P. Kessel, Phys. Rev. E **101**, 023304 (2020).
[9] H. Touchette, Phys. Rep. **478**, 1 (2009).
[10] C. Casert, T. Vieijra, S. Whitelam, and I. Tamblyn, Phys. Rev. Lett. **127**, 120602 (2021).
[11] L. Causer, M. C. Bañuls, and J. P. Garrahan, Phys. Rev. E **103**, 062144 (2021).
[12] G. M. Rotskoff, A. R. Mitchell, and E. Vanden-Eijnden, in *Proceedings of the 2nd Mathematical and Scientific Machine Learning Conference*, Proceedings of Machine Learning Research, Vol. 145, edited by J. Bruna, J. Hesthaven, and L. Zdeborova (PMLR, 2022) pp. 757–780.
[13] J. Yan, H. Touchette, and G. M. Rotskoff, Phys. Rev. E **105**, 024115 (2022).
[14] P. L. Krapivsky, S. Redner, and E. Ben-Naim, *A kinetic view of statistical physics* (Cambridge University Press, 2010).
[15] L. Causer, M. C. Bañuls, and J. P. Garrahan, Phys. Rev. Lett. **128**, 090605 (2022).
[16] R. L. Jack and P. Sollich, J. Phys. A **47**, 015003 (2013).
[17] M. C. Bañuls and J. P. Garrahan, Phys. Rev. Lett. **123**, 200601 (2019).
[18] M. Germain, K. Gregor, I. Murray, and H. Larochelle, in *International conference on machine learning* (PMLR, 2015) pp. 881–889.
[19] D. P. Kingma and J. Ba, arXiv:1412.6980 (2014).
