# Supplementary material for: Learning nonequilibrium statistical mechanics and dynamical phase transitions
Source: Nat Commun. 2024 Feb 6;15:1117. doi: 10.1038/s41467-024-45172-8 (PMC10847122; doi:10.1038/s41467-024-45172-8)
Supplement: Supplementary file 3 — Description of Additional Supplementary Files [file 41467_2024_45172_MOESM3_ESM.pdf]

Description of Additional Supplementary Files

File Name: Supplementary Data 1

Description: A software package for the manuscript "Learning nonequilibrium statistical mechanics and dynamical phase transitions".
